# Targeting the Endoplasmic Reticulum Stress-Linked PERK/GRP78/CHOP Pathway with Magnesium Sulfate Attenuates Chronic-Restraint-Stress-Induced Depression-like Neuropathology in Rats

**DOI:** 10.3390/ph16020300

**Published:** 2023-02-15

**Authors:** Hany H. Arab, Ali Khames, Shuruq E. Alsufyani, Azza A. K. El-Sheikh, Amany M. Gad

**Affiliations:** 1 Department of Pharmacology and Toxicology, College of Pharmacy, Taif University, P.O. Box 11099, Taif 21944, Saudi Arabia; 2 Department of Pharmacology and Toxicology, Faculty of Pharmacy, Sohag University, Sohag 82511, Egypt; 3 Basic Health Sciences Department, College of Medicine, Princess Nourah bint Abdulrahman University, P.O. Box 84428, Riyadh 11671, Saudi Arabia; 4 Department of Pharmacology and Toxicology, Faculty of Pharmacy, Sinai University, Kantara Branch, Ismailia 41636, Egypt; 5 Department of Pharmacology, Egyptian Drug Authority (EDA)—Formerly NODCAR, Giza 12654, Egypt

**Keywords:** magnesium sulfate, stress, neurotransmitters, inflammation, ER stress

## Abstract

Magnesium sulfate has demonstrated marked neuroprotection in eclampsia, hypoxia, stroke, and post-traumatic brain injury rodent models. However, its potential impact against chronic-restraint-stress (CRS)-induced depression-like neuropathology and associated alterations in endoplasmic reticulum (ER) stress have not been adequately examined. The present study aimed to investigate the neuroprotective potential of magnesium sulfate in a rat model of CRS-triggered depression-like behavioral disturbance and the underlying molecular mechanisms. Herein, CRS was induced by placing rats into restraining tubes for 6 h/day for 21 days and the animals were intraperitoneally injected with magnesium sulfate (100 mg/kg/day) during the study period. After stress cessation, the depression-like behavior was examined by the open-field test, sucrose preference test, and forced swimming test. The present data demonstrated that CRS triggered typical depression-like behavioral changes which were confirmed by the Z-normalization scores. Mechanistically, serum circulating corticosterone levels spiked, and the hippocampi of CRS-exposed animals demonstrated a significant decline in serotonin, norepinephrine, and dopamine neurotransmitters. At the molecular level, the hippocampal pro-inflammatory TNF-alpha and IL-1β cytokines and the oxidative stress marker 8-hydroxy-2′-deoxyguanosine (8-HG) increased in stressed animals. In tandem, enhancement of hippocampal ER stress was evidenced by the activation of iNOS/PERK/GRP78/CHOP axis seen by increased protein expression of iNOS, PERK, GRP78, and CHOP signal proteins in the hippocampi of stressed rats. Interestingly, magnesium sulfate administration attenuated the depression-like behavioral outcomes and the histopathological changes in the brain hippocampi. These favorable actions were driven by magnesium sulfate’s counteraction of corticosterone spike, and hippocampal neurotransmitter decline, alongside the attenuation of neuroinflammation, pro-oxidation, and ER stress. In conclusion, the current results suggest the promising neuroprotective/antidepressant actions of magnesium sulfate in CRS by dampening inflammation, ER stress, and the associated PERK/GRP78/CHOP pathway.

## 1. Introduction

Depression is a common mental disorder with serious effects that can disable patients’ daily life activities [1]. Changes in the mental status triggered by the hyperactivity of the hypothalamic-pituitary-adrenal (HPA) axis are hallmarks of the pathogenesis of depressive disorders. Meanwhile, the depletion of monoamines including serotonin, norepinephrine, and dopamine in the hippocampus has been regarded as a crucial underlying mechanism for the neuropathology of depression [2,3]. Stress has been identified as a risk factor for the incidence of major depressive disorder. Several stress models including restraint stress have been introduced in rodents to mimic the occurrence of the depressive-like symptoms, such as dampened locomotor activity and physical state changes [2,4]. In this regard, the chronic restraint stress (CRS) model in rodents (6 h per day for 21 days) has been reported to develop several neurobehavioral abnormalities and clinical depression symptoms similar to the human condition [4]. 

At the cellular and molecular levels, the underlying mechanisms that mediate the pathogenesis of depression-like neuropathology involve an exaggerated oxidative/nitrosative stress and excessive production of pro-inflammatory cytokines, including tumor necrosis factor-alpha (TNF-α) and interleukin 1 beta (IL-1β) in several brain regions, including the hippocampus [2,3,5,6]. The neuropathogenesis of the CRS model also involves the disruption of the HPA axis [2,7]. Notably, structural degeneration and impaired functionality of the hippocampus have been reported as common features in the pathology of depression-like behavior in the CRS model [2,8]. In perspective, CRS-triggered apoptotic cell death and neuronal cell loss in the hippocampus region contribute to the instigation of depression-associated behavioral anomalies [4]. 

**Figure 1:**
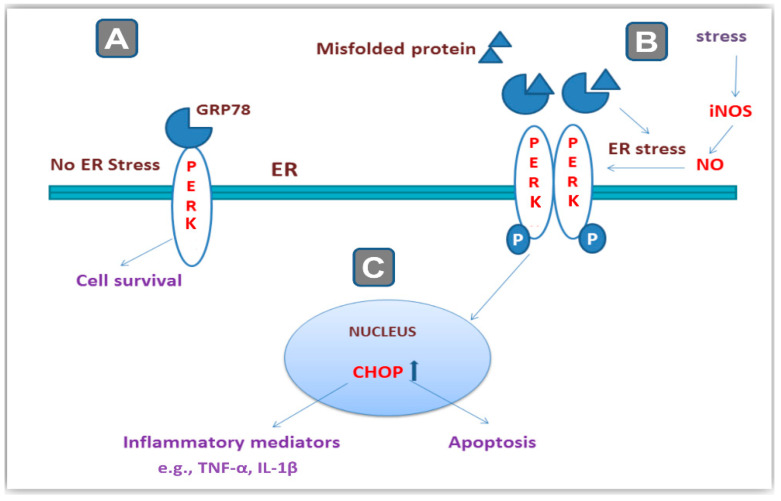
An overview of endoplasmic reticulum (ER) stress and its relationship to cellular stressors and PERK/GRP78/CHOP pathway. (**A**) Under normal conditions when the neurons are not subjected to stressors, the protein kinase RNA-like endoplasmic reticulum kinase (PERK) is kept in an inactive state by binding to glucose-regulated protein 78 (GRP78). (**B**) When cells encounter stressors including excessive nitric oxide (NO) synthesized by inducible nitric oxide synthase (iNOS), the ER stress takes place where misfolded proteins accumulate inside the ER of neurons. As a result, GRP78 is dissociated from PERK which then undergoes phosphorylation/dimerization, triggering its activation. (**C**) The later event upregulates the protein expression of the C/EBP-homologous protein (CHOP) signal culminating in several pro-apoptotic responses and neuronal cell loss alongside the increased synthesis of multiple pro-inflammatory cytokines, such as tumor necrosis factor-alpha (TNF-α) and interleukin 1 beta (IL-1β).

Ample evidence revealed the involvement of the endoplasmic reticulum (ER) stress in the pathogenesis of CRS- and chronic-social-defeat-induced depression-like behavior [3,4]. In neuronal cells, the ER is a crucial cellular organelle for the proper synthesis and folding of proteins. Multiple pathological conditions, including stress, hypoxia, and oxidative stress have been characterized to elicit ER stress where misfolding of the cellular proteins prevails [9]. Meanwhile, inducible nitric oxide synthase (iNOS) has been described as an inducer of ER stress by overshooting of nitric oxide radicals [10]. In ER stress, cells respond by triggering the unfolded protein response (UPR) which is an adaptive response that aims at promoting homeostasis in the ER. In this context, correct protein folding is restored by the molecular chaperones including 78-kDa glucose-regulated protein 78 (GRP78) and 94-kDa glucose-regulated protein (GRP94). In non-stressed cells, the protein kinase RNA-like endoplasmic reticulum kinase (PERK) is kept in an inactive state through its binding to GRP78 [2,3,4] (Figure 1). When the extent of ER stress is overwhelming, cells respond by upregulating C/EBP-homologous protein (CHOP) and other factors that trigger neuronal cell apoptosis. In this context, GRP78 is dissociated from PERK which then undergoes an auto-phosphorylation/dimerization triggering the activation of PERK. The later event upregulates the protein expression of CHOP culminating in several pro-apoptotic responses and neuronal loss [3,4,11]. Activated PERK has been reported to induce the synthesis of multiple pro-inflammatory mediators, such as IL-1β [12]. Hence, PERK/GRP78/CHOP, a signaling cascade that mediates ER stress, plays a crucial role in the pathogenesis of stress-related behavioral outcome alterations.

In clinical practice, diverse adverse effects have been linked to the use of several antidepressant medications, especially when used in large doses or for long durations [13]. Clinical trials have also demonstrated the ineffectiveness of some of the current treatments against depression [14]. Hence, the quest for alternative agents with potential antidepressant actions is warranted. Magnesium sulfate has been commonly used for the control of severe eclampsia and life-threatening seizures during pregnancy in humans [15]. Moreover, it demonstrated marked neuroprotection in the preclinical models of eclampsia [16] and acute stroke [17] by lowering pro-inflammatory cytokine levels. Magnesium sulfate has also been reported to counteract neuronal injury by curbing oxidative stress [18]. In ischemic rodent models, magnesium sulfate has been reported to dampen neuronal apoptosis and improve neuronal activity, culminating in lowered cerebral infarct volume [16]. Likewise, the cerebral contusion in vivo models revealed the efficacy of magnesium sulfate for combating neuronal cell death and edema [16,18]. In addition, it has also been reported that magnesium sulfate is competent to lessen pain in experimental animal models [19]. Of note, magnesium sulfate has been characterized to gain access to brain regions by penetrating the blood-brain barrier, particularly under brain pathological conditions [18]. In the current work, we hypothesized that magnesium sulfate may exert beneficial neuroprotection against CRS-triggered depression-like behavioral disturbance. Hence, the present work aimed at exploring the neuroprotective potential of magnesium sulfate to mitigate CRS-triggered depression-like behavioral disturbance and the underlying molecular mechanisms, including neuroinflammation and ER stress.

## 2. Results

### 2.1. Chronic-Restraint-Stress (CRS)-Induced Depression-like Behavioral Deficits Were Mitigated by Magnesium Sulfate

**Figure 2:**
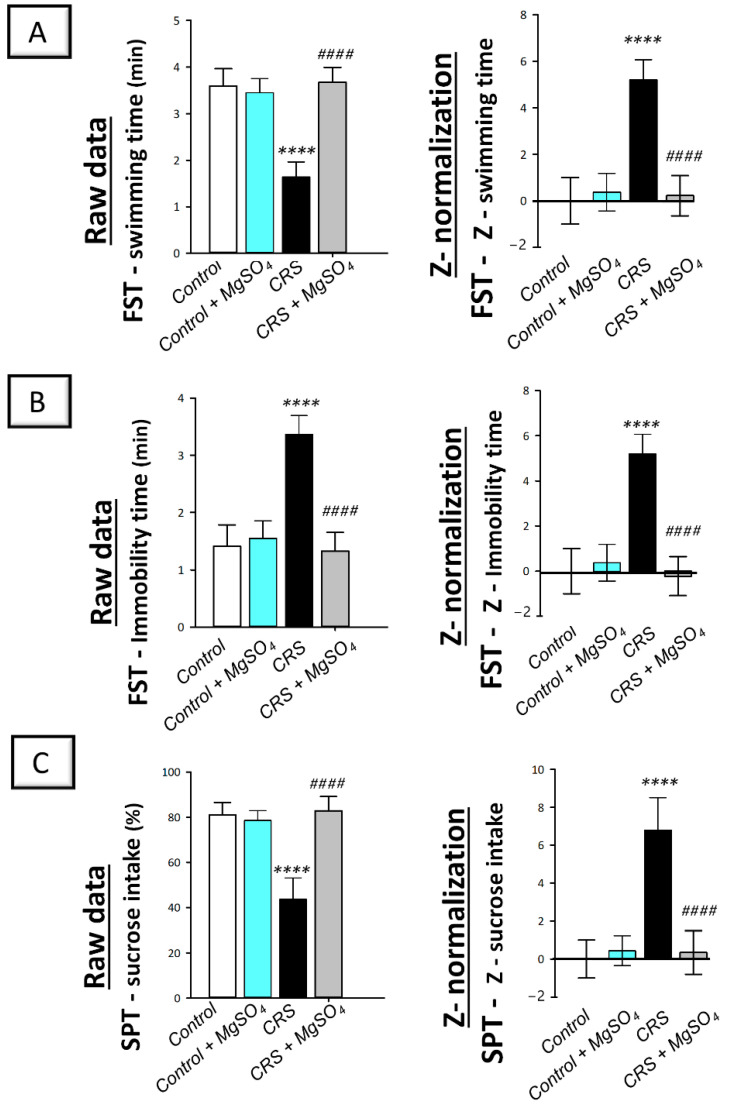
Effect of magnesium sulfate on the depression-like behavioral deficits triggered by chronic restraint stress (CRS) in the forced swimming test (FST) and sucrose preference test (SPT) in rats. Administration of magnesium sulfate attenuated the depression-like behavioral deficits that were invoked by CRS. The beneficial effects of magnesium sulfate were demonstrated by the increased swimming time [F (3, 20) = 50.79, *p* < 0.0001] (**A**) and lowered immobility time [F (3, 20) = 50.80, *p* < 0.0001] (**B**) in the FST alongside the increased sucrose intake percentage [F (3, 20) = 46.77, *p* < 0.0001] in the SPT (**C**). Calculation of Z-values was described in the material and methods Section 4.5.4. using the control group as the baseline. Z-values are expressed as the mean of 6 Z-scores ± standard deviation (S.D.). Statistical analysis was applied using one-way ANOVA followed by Tukey’s post-hoc test. Significant differences versus control animals are denoted by **** p < 0.001. Significant differences versus CRS group are denoted by ^#*###*^ *p* < 0.001. CRS, chronic restraint stress; MgSO_4_, magnesium sulfate.

The depression-like behavior triggered by CRS was examined by the forced swimming test (FST) and sucrose preference test (SPT) in rats. As illustrated in Figure 2A,B (left panels), significant differences were detected among experimental groups in the swimming time (F (3, 20) = 50.79, *p* < 0.0001) and immobility time (F (3, 20) = 50.80, *p* < 0.0001) in the FST. Moreover, a significant difference was detected among experimental groups in the sucrose intake percentage (F (3, 20) = 46.77, *p* < 0.0001) in the SPT (Figure 2C; left panel). In this regard, CRS prompted behavioral despair that was revealed by a significant decrease in the swimming time by 54.4% (*p* < 0.0001) together with a significant increase in the immobility time by 138.4% (*p* < 0.0001) in the FST (*p* < 0.0001) versus the control animals. In the SPT, CRS triggered a significant decrease in sucrose intake percentage by 46.1% (*p* < 0.0001) versus the control animals. Magnesium sulfate (100 mg/kg; i.p.) administration to CRS-exposed animals counteracted these behavioral deficits as seen by a significant increase in the swimming time by 124.3% (*p* < 0.0001) and a significant decrease in the immobility time by 60.5% (*p* < 0.0001) in the FST. Moreover, magnesium sulfate resulted in a significant increase in sucrose intake percentage by 89.9% (*p* < 0.0001) in the SPT versus the CRS group.

Notably, the beneficial effects of magnesium sulfate on CRS-induced depression-like behavior were confirmed by Z-score normalization which imparts robust and reliable data (Figure 2; right panels) [20]. The Z -score normalization transforms the raw data values to the number of standard deviations from the control mean. As illustrated in Figure 2A,B (right panels), CRS triggered a significant difference in the Z-values of swimming time and immobility time by 519.9% (*p* < 0.0001) and by 520% (*p* < 0.0001), respectively, in the FST and the Z-values of sucrose consumption by 679.1% (*p* < 0.0001) in the SPT versus the control animals. These changes were significantly counteracted by magnesium sulfate administration to CRS-exposed animals. Together, these data depict the competence of magnesium sulfate for mitigating the depression-like behavioral deficits driven by CRS in rats. 

### 2.2. CRS-Induced Decline in the Locomotor Activity Was Counteracted by Magnesium Sulfate

**Figure 3:**
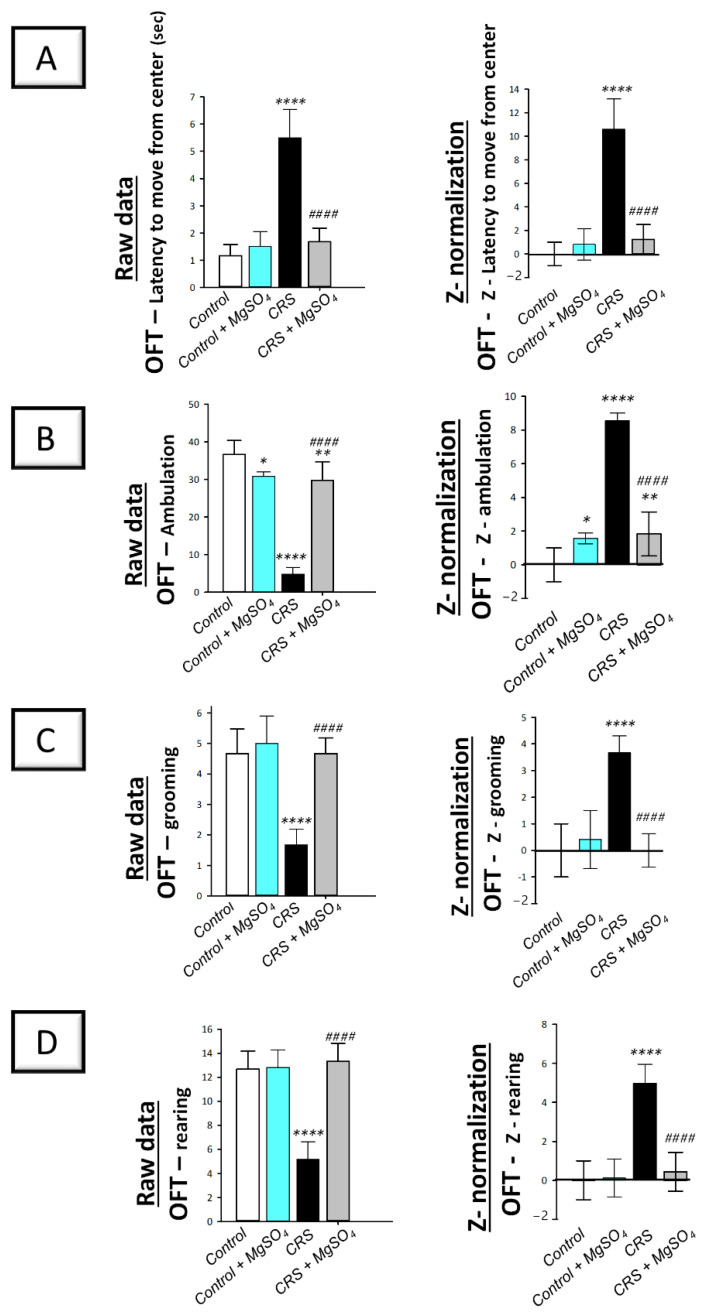
Effect of magnesium sulfate on the depression-like behavioral changes triggered by chronic restraint stress (CRS) in the open field test (OFT) in rats. Administration of magnesium sulfate attenuated the depression-associated decline in locomotor activity that was invoked by CRS. The beneficial effects of magnesium sulfate were demonstrated by the decreased latency time to move from the center [F (3, 20) = 54.39, *p* < 0.0001] (**A**) alongside the increased ambulation [F (3, 20) = 114.2, *p* < 0.0001] (**B**), grooming [F (3, 20) = 29.33, *p* < 0.0001] (**C**), and rearing [F (3, 20) = 41.15, *p* < 0.0001] (**D**) in the OFT. Calculation of Z-values was described in the material and methods Section 4.5.4. using the control group as the baseline. Z-values are expressed as the mean of 6 Z-scores ± standard deviation (S.D.). Statistical analysis was applied using one-way ANOVA followed by Tukey’s post-hoc test. Significant differences versus control animals are denoted by * p < 0.05, ** p < 0.01, or **** p < 0.001. Significant differences versus CRS group are denoted by ^#*###*^ *p* < 0.001. CRS, chronic restraint stress; MgSO_4_, magnesium sulfate.

The depression-like behavior triggered by CRS was further studied by the open field test (OFT) in rats. As illustrated in Figure 3 (left panels), significant differences were detected among experimental groups in the latency time to move from the central square [F (3, 20) = 54.39, *p* < 0.0001], ambulation [F (3, 20) = 114.2, *p* < 0.0001], grooming [F (3, 20) = 29.33, *p* < 0.0001], and rearing [F (3, 20) = 41.15, *p* < 0.0001] in the OFT. In this regard, CRS prompted behavioral despair that was associated with diminished locomotor activity seen by a significant increase in the latency time in the OFT by 371.3% (*p* < 0.0001) versus the control animals. In the same context, CRS prompted a significant decrease in the ambulation by 86.6% (*p* < 0.0001), grooming by 64.3% (*p* < 0.0001), and rearing by 59.2% (*p* < 0.0001) versus the control animals (Figure 3B–D; left panels). Magnesium sulfate (100 mg/kg; i.p.) administration to CRS-exposed animals counteracted the decline in locomotor activity as seen by a significant decrease in the latency time by 69.7% (*p* < 0.0001) alongside a significant increase in ambulation by 517.2% (*p* < 0.0001), grooming by 180% (*p* < 0.0001), and rearing by 158% (*p* < 0.0001) versus the CRS group.

Notably, the beneficial effects of magnesium sulfate on CRS-induced decline in locomotor activity were confirmed by Z -score normalization. As illustrated in Figure 3 (right panels), CRS triggered a significant difference in the Z-values of latency time, ambulation, grooming, and rearing by 1061% (*p* < 0.0001), 854.8% (*p* < 0.0001), 367.4% (*p* < 0.0001), and 498.1% (*p* < 0.0001), respectively, in the OFT versus the control animals. These changes were significantly counteracted by magnesium sulfate administration to CRS-exposed animals. Together, these data depict the competence of magnesium sulfate in improving the depression-associated decline in locomotor activity that was driven by CRS in rats. 

### 2.3. CRS-Induced Spike in Serum Corticosterone Was Attenuated by Magnesium Sulfate

**Figure 4:**
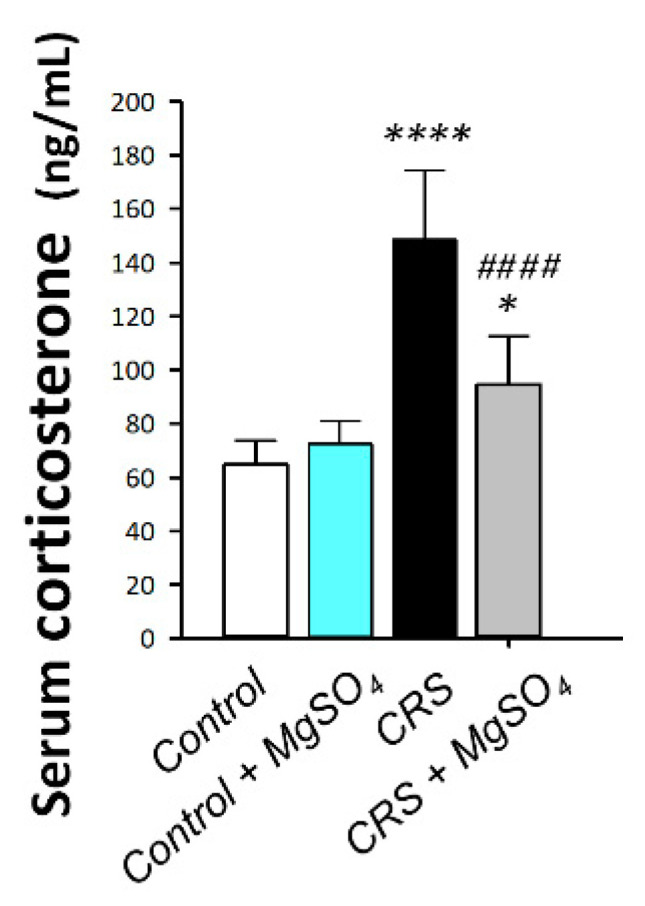
Effect of magnesium sulfate on serum corticosterone levels in rats exposed to chronic restraint stress (CRS). Administration of magnesium sulfate attenuated the increased serum corticosterone levels invoked by CRS [F (3, 20) = 30.08, *p* < 0.0001]. Values are expressed as the mean of 6 rats ± standard deviation (S.D.). Statistical analysis was applied using one-way ANOVA followed by Tukey’s post-hoc test. Significant differences versus control animals are denoted by * p < 0.05, or **** p < 0.001. Significant differences versus CRS group are denoted by ^#*###*^ *p* < 0.001. CRS, chronic restraint stress; MgSO_4_, magnesium sulfate.

The hypothalamic-pituitary-adrenal (HPA) axis was explored by measuring serum corticosterone levels in rats, the principal glucocorticoid in rodents [21]. As illustrated in Figure 4, a significant difference was detected among experimental groups in serum corticosterone levels [F (3, 20) = 30.08, *p* < 0.0001]. In this regard, CRS provoked a significant increase in serum corticosterone level by 129.5% (*p* < 0.0001) versus the control animals. Magnesium sulfate (100 mg/kg; i.p.) administration to CRS-exposed animals counteracted the stress response in the animals as seen by a significant decrease in serum corticosterone by 36.4% (*p* < 0.001) versus the CRS group. These findings illustrated the competence of magnesium sulfate for dampening the stress response in animals exposed to CRS by lowering serum corticosterone. 

### 2.4. CRS-Induced Decline in Hippocampal Monoamine Neurotransmitters Was Counteracted by Magnesium Sulfate

**Figure 5:**
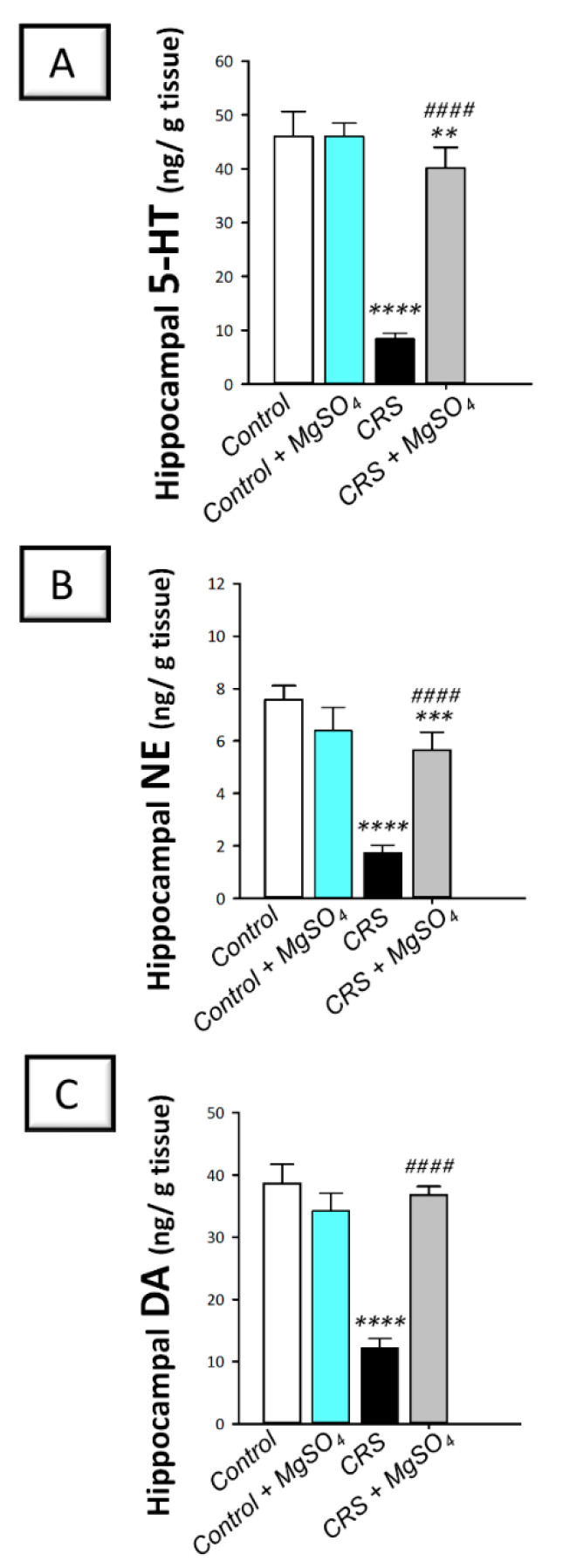
Effect of magnesium sulfate on monoamine neurotransmitter levels in the hippocampi of rats exposed to chronic restraint stress (CRS). Administration of magnesium sulfate counteracted the depletion of serotonin [F (3, 20) = 274.0, *p* < 0.0001] (**A**), norepinephrine [F (3, 20) = 95.76, *p* < 0.0001] (**B**), and dopamine [F (3, 20) = 167.7, *p* < 0.0001] (**C**) in the hippocampi of rats exposed to CRS. Values are expressed as the mean of 6 rats ± standard deviation (S.D.). Statistical analysis was applied using one-way ANOVA followed by Tukey’s post-hoc test. Significant differences versus control animals are denoted by ** p < 0.01, *** p < 0.001, or **** p < 0.001. Significant differences versus CRS group are denoted by ^#*###*^ *p* < 0.001. CRS, chronic restraint stress; MgSO_4_, magnesium sulfate.

As illustrated in Figure 5A–C, significant differences were detected among experimental groups in the hippocampal levels of serotonin [F (3, 20) = 274.0, *p* < 0.0001], norepinephrine [F (3, 20) = 95.76, *p* < 0.0001], and dopamine [F (3, 20) = 167.7, *p* < 0.0001]. In this regard, CRS provoked a significant decline in serotonin (5-HT), norepinephrine (NE), and dopamine (DA) levels by 81.60% (*p* < 0.0001), 77.1% (*p* < 0.0001), and 68.4% (*p* < 0.0001), respectively, versus the control animals. Magnesium sulfate (100 mg/kg; i.p.) administration to CRS-exposed animals enhanced the hippocampal levels of these neurotransmitters as seen by a significant increase in the 3 monoamine neurotransmitters by 374.4% (*p* < 0.0001), 226.6% (*p* < 0.0001), and 201.7% (*p* < 0.0001), respectively, versus the CRS group. These data depict that augmentation of hippocampal monoamine neurotransmitters is, at least partly, involved in the mitigation of CRS-triggered depression-like behavior in rats.

### 2.5. CRS-Induced Increase in Hippocampal Pro-Inflammatory Cytokines Was Curbed by Magnesium Sulfate

**Figure 6:**
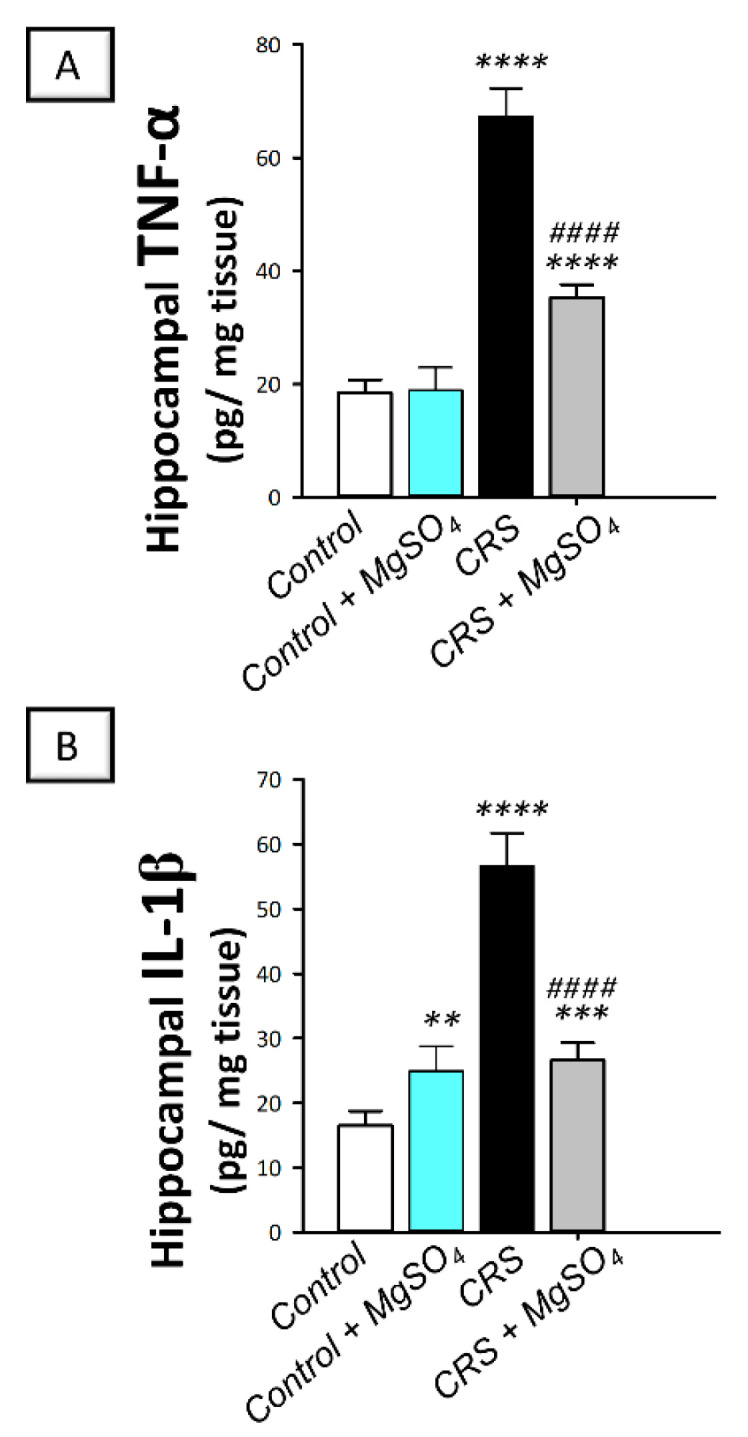
Effect of magnesium sulfate on the pro-inflammatory cytokine levels in the hippocampi of rats exposed to chronic restraint stress (CRS). Administration of magnesium sulfate suppressed the increased levels of tumor necrosis factor-alpha (TNF-α; (**A**)) [F (3, 20) = 244.6, *p* < 0.0001] and interleukin 1 beta (IL-1β; (**B**)) [F (3, 20) = 159.2, *p* < 0.0001] in the hippocampi of rats exposed to CRS. Values are expressed as the mean of 6 rats ± standard deviation (S.D.). Statistical analysis was applied using one-way ANOVA followed by Tukey’s post-hoc test. Significant differences versus control animals are denoted by ** p < 0.01, *** p < 0.001, or **** p < 0.001. Significant differences versus CRS group are denoted by ^#*###*^ *p* < 0.001. CRS, chronic restraint stress; MgSO_4_, magnesium sulfate.

As illustrated in Figure 6A,B, significant differences were detected among experimental groups in the hippocampal levels of tumor necrosis factor-alpha (TNF-α) [F (3, 20) = 244.6, *p* < 0.0001] and interleukin 1 beta (IL-1β) [F (3, 20) = 159.2, *p* < 0.0001]. In this regard, CRS provoked a severe proinflammatory response in the hippocampi of rats exposed to CRS. This was demonstrated by a significant increase in TNF-α and IL-1β levels by 265.3% (*p* < 0.0001) and 243.8% (*p* < 0.0001), respectively, versus the control animals. Magnesium sulfate (100 mg/kg; i.p.) administration to CRS-exposed animals attenuated the hippocampal pro-inflammatory events as seen by a significant decrease in TNF-α and IL-1β by 47.5% (*p* < 0.0001) and 53.1% (*p* < 0.0001), respectively, versus the CRS group. These findings demonstrate that attenuation of hippocampal pro-inflammatory cytokines is, at least partly, involved in the amelioration of CRS-triggered depression-like behavior in rats.

### 2.6. CRS-Induced Increase in the Hippocampal Oxidative/Nitrosative Stress Markers Was Attenuated by Magnesium Sulfate

**Figure 7:**
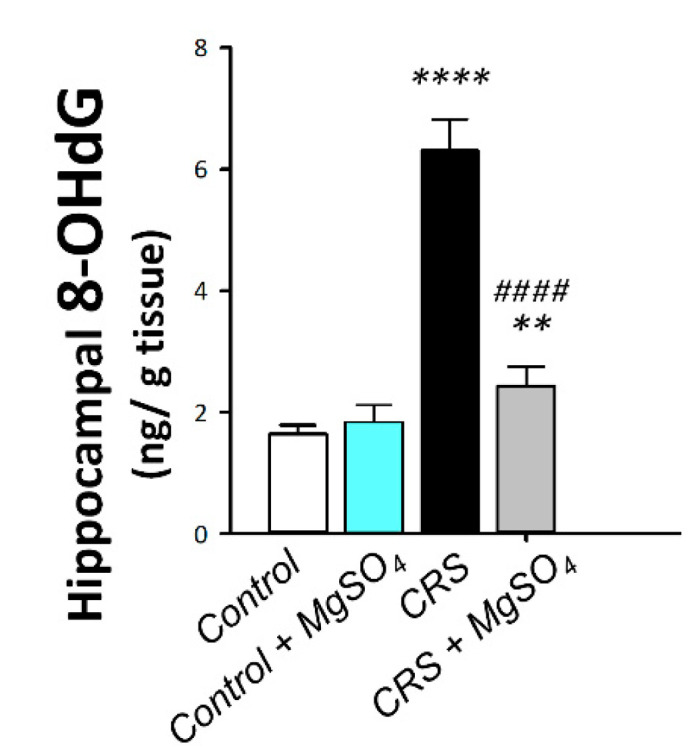
Effect of magnesium sulfate on the oxidative stress marker 8-hydroxy-2′-deoxyguanosine (8-OhdG) in the hippocampi of rats exposed to chronic restraint stress (CRS). Administration of magnesium sulfate lowered the increased levels of 8-OHdG [F (3, 20) = 250.7, *p* < 0.0001] in the hippocampi of rats exposed to CRS. Values are expressed as the mean of 6 rats ± standard deviation (S.D.). Statistical analysis was applied using one-way ANOVA followed by Tukey’s post-hoc test. Significant differences versus control animals are denoted by ** p < 0.01, or **** p < 0.001. Significant differences versus CRS group are denoted by ^#*###*^ *p* < 0.001. CRS, chronic restraint stress; MgSO_4_, magnesium sulfate.

**Figure 8:**
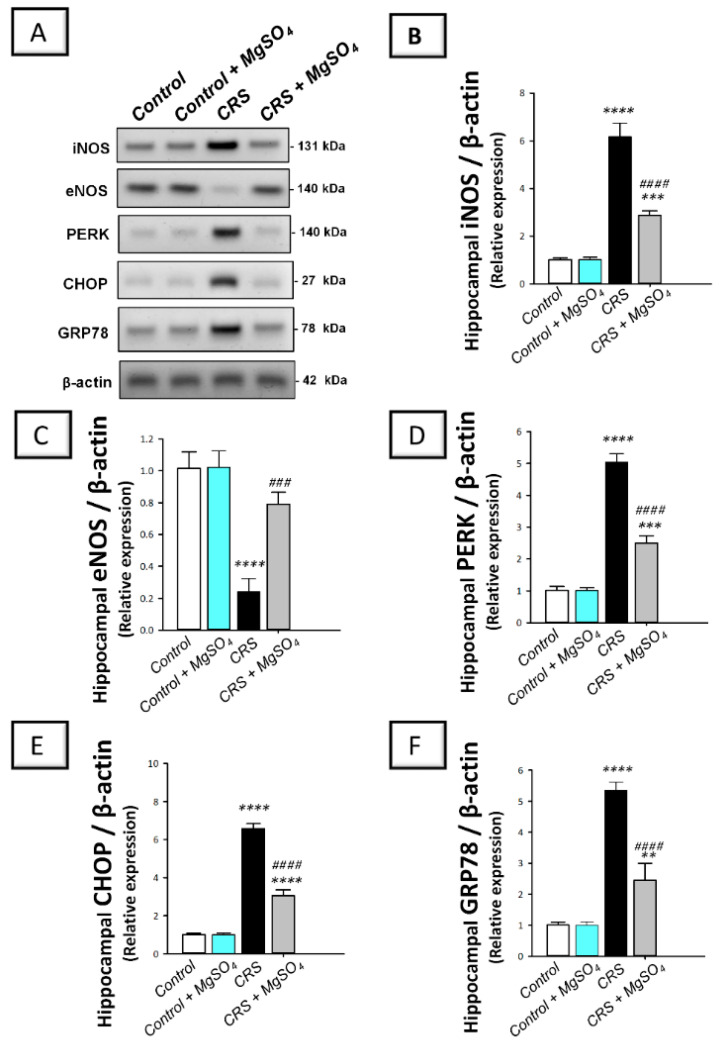
Effect of magnesium sulfate on endoplasmic reticulum (ER) stress and the associated PERK/GRP78/CHOP pathway in the hippocampi of rats exposed to chronic restraint stress (CRS). The protein expression of the signals involved in the ER-stress-associated PERK/GRP78/CHOP pathway was detected by Western blotting (**A**). Administration of magnesium sulfate counteracted the ER stress response that was switched on by CRS. The beneficial effects of magnesium sulfate were demonstrated by the decreased protein expression of the inducible nitric oxide synthase (iNOS, (**B**)) [F (3, 8) = 215.5, *p* < 0.0001] and augmented protein expression of the endothelial nitric oxide synthase (eNOS, (**C**)) [F (3, 8) = 45.87, *p* < 0.0001] in the hippocampi of rats exposed to CRS. Moreover, magnesium sulfate downregulated the protein expression of the protein kinase RNA-like endoplasmic reticulum kinase (PERK, (**D**)) [F (3, 8) = 232.7, *p* < 0.0001], C/EBP-homologous protein (CHOP, (**E**)) [F (3, 8) = 935, *p* < 0.0001], and glucose-regulated protein 78-kDa (GRP78, (**F**)) [F (3, 8) = 120.2, *p* < 0.0001] in the hippocampi of rats exposed to CRS. The quantification values of immunoblotting are expressed as the mean of 3 independent values ± standard deviation (S.D.). Statistical analysis was applied using one-way ANOVA followed by Tukey’s post-hoc test. Significant differences versus control animals are denoted by ** p < 0.01, *** p < 0.001, or **** p < 0.001. Significant differences versus CRS group are denoted by *^###^ p* < 0.001, or ^#*###*^ *p* < 0.001. CRS, chronic restraint stress; MgSO_4_, magnesium sulfate.

As illustrated in Figure 7, a significant difference was detected among experimental groups in the hippocampal levels of the pro-oxidant marker 8-hydroxy-2′-deoxyguanosine (8-OHdG) [F (3, 20) = 250.7, *p* < 0.0001]. In this regard, CRS prompted an exaggerated oxidative stress response in the hippocampi of rats exposed to CRS. This was demonstrated by a significant increase in the levels of 8-OhdG by 284.8% (*p* < 0.0001) versus the control animals. In the same regard, the nitrosative stress was explored by determining the protein expression of the inducible nitric oxide synthase (iNOS) pro-oxidant signal together with the endothelial nitric oxide synthase (eNOS) neuroprotective signal in the hippocampi of rats. As demonstrated in Figure 8B,C, significant differences were detected among experimental groups in the protein expression levels of iNOS [F (3, 8) = 215.5, *p* < 0.0001] and eNOS [F (3, 8) = 45.87, *p* < 0.0001] in the hippocampi of rats. In this regard, CRS prompted a significant increase in the protein expression of iNOS by 509.9% (*p* < 0.0001) and a significant decrease in the protein expression of eNOS by 76.4% (*p* < 0.0001) versus the control animals. Magnesium sulfate (100 mg/kg; i.p.) administration to CRS-exposed animals significantly lowered the 8-OhdG levels and the protein expression of iNOS by 61.5% (*p* < 0.0001) and 53.2% (*p* < 0.0001), respectively, versus the CRS group. Moreover, magnesium sulfate significantly increased eNOS protein expression by 230% (*p* < 0.001) versus the CRS group. These findings demonstrate that the attenuation of hippocampal oxidative/nitrosative stress is, at least partly, involved in the mitigation of CRS-triggered depression-like behavior in rats.

### 2.7. CRS-Induced Hippocampal Activation of PERK/GRP78/CHOP Pathway Was Curtailed by Magnesium Sulfate

As illustrated in Figure 8D–F, significant differences were detected among experimental groups in the protein expression levels of the protein kinase RNA-like endoplasmic reticulum kinase (PERK) [F (3, 8) = 232.7, *p* < 0.0001], C/EBP-homologous protein (CHOP) [F (3, 8) = 935, *p* < 0.0001], and glucose-regulated protein 78-kDa (GRP78) [F (3, 8) = 120.2, *p* < 0.0001] in the hippocampi of rats. In this regard, CRS prompted the activation of the ER-stress-linked PERK/GRP78/CHOP pathway in the hippocampi of rats exposed to CRS. This was demonstrated by a significant increase in the protein expression of PERK, CHOP, and GRP78 by 396.1% (*p* < 0.0001), 552.8% (*p* < 0.0001), and 429.6% (*p* < 0.0001), respectively, versus the control animals. Magnesium sulfate (100 mg/kg; i.p.) administration to CRS-exposed animals inhibited the signaling of PERK/GRP78/CHOP axis. This was demonstrated by a significant decrease in the protein expression of PERK, CHOP, and GRP78 by 50.4% (*p* < 0.0001), 53.6% (*p* < 0.0001), and 54.3% (*p* < 0.0001), respectively, versus the CRS group. These findings demonstrate that the inhibition of PERK/GRP78/CHOP-associated ER stress response by magnesium sulfate, is at least partly, involved in the improvement of CRS-triggered depression-like behavior in rats.

### 2.8. CRS-Induced Histopathological Changes in Brain Regions Were Attenuated by Magnesium Sulfate

**Figure 9:**
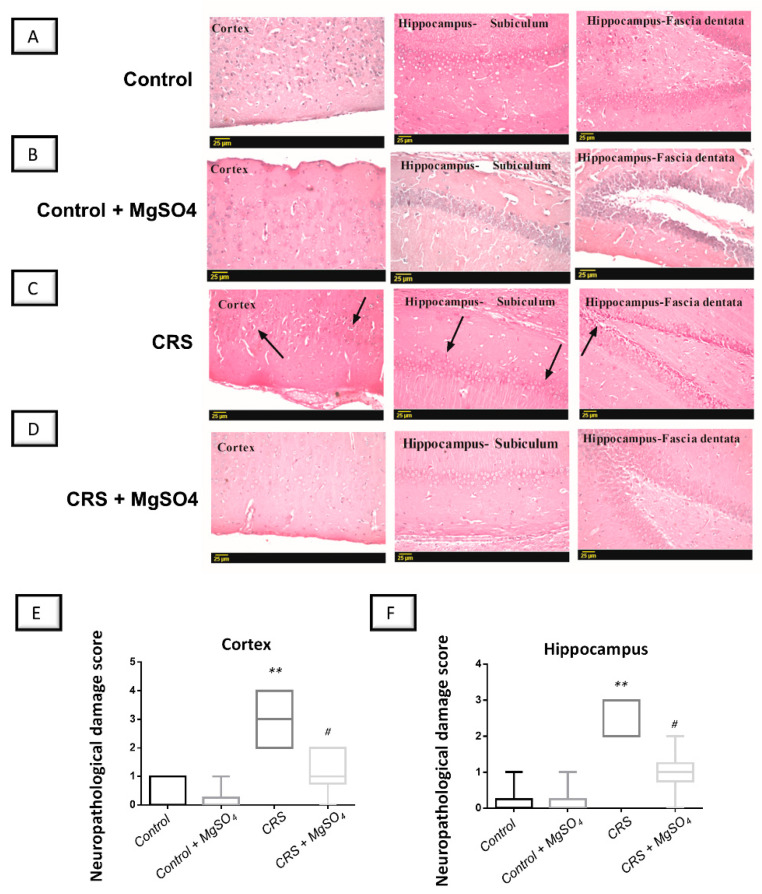
Effect of magnesium sulfate on brain histological alterations in rats exposed to chronic restraint stress (CRS). The brain sections were stained with hematoxylin/eosin (H/E) and were examined under light microscope (Representative micrographs; scale bar = 25 µm). Administration of magnesium sulfate counteracted the histopathological changes by CRS in the hippocampus and cortex regions in rats’ brains. (**A**,**B**) Microscopic investigation of brain sections revealed a normal histological structure in the control and magnesium-sulfate-treated group in the cerebral cortex alongside fascia dentata and hilus of the hippocampus. (**C**) In the CRS group, evident nuclear pyknosis (arrow) and degeneration were detected in the neurons located in the cortices and hippocampi of animals. (**D**) These alterations were attenuated by magnesium sulfate administration to the CRS-exposed rats. (**E**,**F**) The histopathological damage in the cortices and hippocampi of the animals, respectively, was quantified as the neuropathological damage scores. Administration of magnesium sulfate lowered the neuropathological damage scores in the cortices [H (3, 20) = 16.89, *p* = 0.0007] and hippocampi [H (3, 20) = 17.87, *p* = 0.0005] of rats exposed to CRS. The values of neuropathological scores were represented as the median with the interquartile range (*n* = 6). Statistical analysis was applied using the Kruskal–Wallis test followed by Dunn’s multiple comparison post-test. Significant differences versus control animals are denoted by ** p < 0.01. Significant differences versus CRS group are denoted by *^#^ p* < 0.05. CRS, chronic restraint stress; MgSO_4_, magnesium sulfate. CRS, chronic restraint stress; MgSO_4_, magnesium sulfate.

Ample evidence exists that marked neuropathological and degenerative changes were detected in the cortices and hippocampi of rodents with stress-triggered depression [22,23]. Hence, the present work investigated the histopathological alterations in the cortices and hippocampi of rats. As illustrated in Figure 9, CRS elicited marked histopathological alterations in the cerebral cortex and hippocampus with evident nuclear pyknosis and degeneration in neurons located in these brain regions. The histopathological damage was quantified as the neuropathological damage scores. As illustrated in Figure 9E,F, a significant difference was detected among experimental groups in the neuropathological damage scores in the cortices [H (3, 20) = 16.89, *p* = 0.0007] and hippocampi of animals [H (3, 20) = 17.87, *p* = 0.0005]. Notably, the damage scores were attenuated by magnesium sulfate administration to the CRS-exposed rats in the cortices and hippocampi of animals by 61.1% (*p* < 0.05) and 62.5% (*p* < 0.05), respectively, versus the CRS group. These data demonstrate the competence of magnesium sulfate to attenuate brain pathological changes at the microscopic level in CRS-exposed animals. 

## 3. Discussion

**Figure 10:**
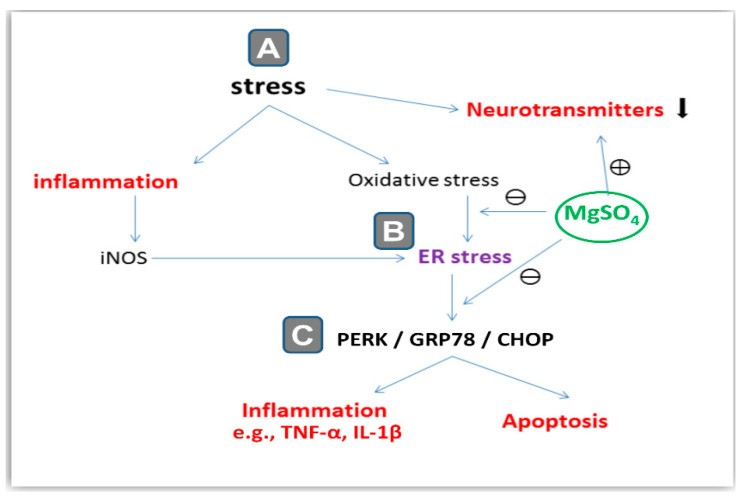
An overview of the underlying mechanisms that explain the observed antidepressant effect of magnesium sulfate against stress-induced depression-like behavior in rats. (**A**) Exposure to chronic restraint stress (CRS) resulted in the depletion of neurotransmitters, exaggerated oxidative stress, and inflammation in the hippocampi of rats. (**B**) The excessive pro-oxidant/pro-inflammatory responses triggered the endoplasmic reticulum (ER) stress with an accumulation of misfolded proteins in the ER. (**C**) The later event was marked by activation of PERK/GRP78/CHOP pathway which culminates in neuronal inflammation and apoptosis. Interestingly, magnesium sulfate inhibited the ER stress and the linked activation of iNOS/PERK/GRP78/CHOP pathway and suppressed the pro-oxidant/pro-inflammatory signals. These actions prompted the augmentation of the monoamine neurotransmitters in the hippocampi of rats, culminating in improvement of the depression-like behavioral deficits triggered by CRS. ⊕ indicates stimulation while ⊖ indicates inhibition. PERK, protein kinase RNA-like endoplasmic reticulum kinase; GRP78, glucose-regulated protein 78-kDa; CHOP, C/EBP-homologous protein.

In the current study, magnesium sulfate intraperitoneal administration improved the depression-like behavior triggered by chronic restraint stress (CRS) in rats. At the cellular and molecular levels, the neuroprotective impact of magnesium sulfate was mediated by inhibiting hippocampal ER stress and the linked suppression of iNOS/PERK/GRP78/CHOP pathway alongside dampening the pro-oxidant/pro-inflammatory signals (Figure 10).

The neuropathology of depression is explained on the basis of several theories characterizing abnormalities in the hypothalamic-pituitary-adrenal (HPA) axis and deficiency of hippocampal monoamine neurotransmitters [24]. Ample evidence exists that CRS-induced depression in rodents mimics the development of clinical depression symptoms in humans. In perspective, CRS in animals prompts behavioral despair evidenced by reduced sucrose intake, decrease in responsiveness to rewarding stimuli, and locomotor activity deficit [2,3,25]. Consistently, the present findings revealed that CRS prompted depression-like behavior seen by a decline in sucrose consumption in the sucrose preference test, lowered swimming time in the forced swimming test, and diminished locomotor activity in the open-field test. Mechanistically, chronic exposure to stress has been reported to trigger the HPA axis hyperactivity, culminating in elevated serum corticosterone levels and associated behavioral deficits [26]; events that are in line with the present findings. In the hippocampus, the interaction between the HPA axis and the monoamine system plays a crucial role in the pathogenesis of depression through the depletion of monoamine neurotransmitters [2,7]. Conceptually, the depression-like behavioral anomalies are linked to the lowered hippocampal levels of serotonin, norepinephrine, and dopamine neurotransmitters [27], which is in accord with the current results. The lowered hippocampal neurotransmitter levels are likely instigated by the rapid breakdown of monoamines in response to stress and/or their dampened synthesis due to loss of monoamines neurons [28]. In the same regard, increased glucocorticoid levels have been reported to curb norepinephrine release [29]. Herein, magnesium sulfate diminished serum corticosterone spike and augmented hippocampal monoamine neurotransmitters, culminating in improved behavioral outcomes. In fact, interventions, such as selective serotonin reuptake inhibitors (SSRIs) and monoamine oxidase inhibitors (MAOIs), that can augment the monoamine neurotransmitters in several brain regions including the hippocampus have been proven as competent tools for improving the symptoms of depression. Notably, magnesium has been proven to enhance the interaction between serotonin and its receptors, thereby improving the neuronal transmission of the serotoninergic signal. Moreover, the observed decline in serum corticosterone level is likely mediated by the reported magnesium-induced suppression of calcium-mediated glucocorticoid exocytosis release [30]. 

Studies have demonstrated that CRS prompts an exaggerated oxidative stress response in several brain regions of rodents [2,3,5]. In line with this concept, the present data revealed increased levels of the pro-oxidant 8-OHdG and the protein expression of iNOS in the hippocampi of the animals exposed to CRS. In fact, the enhanced oxidative stress is likely linked to the increased glucocorticoid levels that trigger excessive reactive oxygen species (ROS) production [31]. In the same context, the present findings demonstrated an enhanced pro-inflammatory response in the hippocampi of animals as seen by increased levels of TNF-α and IL-1β cytokines. In fact, microglia and astrocytes have been demonstrated to express increased levels of several pro-inflammatory cytokines, favoring neuronal hyperexcitability [16]. The observed results concur with the previous studies [3,4,16] which revealed that psychological stressors including CRS augment the production of pro-inflammatory cytokines in the brain hippocampus. The increase in inflammatory markers in the central nervous system is associated with an enhanced production of free radicals and depletion of neurotransmitters, such as dopamine and norepinephrine [32].Oxidative stress and neuroinflammation have been reported to play crucial roles in the neuropathology of depression [3,33]. In perspective, the marked increase in pro-oxidants and pro-inflammatory cytokines in the brain hippocampus in response to the continuous stress creates a vicious cycle that further disrupts the HPA axis, culminating in neuronal cell death and depression-like behavioral outcomes [4]. Herein, magnesium sulfate blocked the vicious cycle by attenuating the pro-inflammatory cytokines and oxidative stress markers, culminating in improved behavioral events. In fact, abrogation of pro-inflammatory cytokine production and associated neuronal loss in the hippocampus mediated magnesium sulfate’s protection against eclampsia-like seizure in rats [16]. The marked anti-inflammatory features of magnesium sulfate have been also demonstrated in experimental models of Alzheimer’s disease [34], brain injury in fetal mice [35], and cerebral palsy [36]. In the current study, magnesium sulfate exerted notable antioxidant actions by dampening hippocampal 8-OHdG levels and iNOS protein expression. This is in accord with the reported ROS-lowering features and mitochondrial-preserving actions of magnesium sulfate in the brain of animals with hypoxic ischemia [37]. In a rat model of doxorubicin-evoked cardiotoxicity, magnesium sulfate has been reported to protect against cardiac injury by augmenting antioxidant moieties in myocytes [38].

ER stress has been proven as a crucial player in the pathogenesis of depression, bipolar disorder, and stress-induced cognitive decline [2,3,4]. Basically, the ER is an essential cellular organelle that plays a key role in proper protein folding. Several destructive stimuli/pathological conditions including stress, hypoglycemia, hypoxia, oxidative stress, and pro-inflammatory events have been reported to impair ER function culminating in ER stress and accumulation of misfolded proteins [3,11]. These events trigger a process known as the unfolded protein response (UPR), a self-protecting signaling pathway that aims at addressing the accumulation of misfolded proteins by enhancing the transcriptional synthesis of ER chaperones such as GRP78. Yet, when severe ER stress is encountered by neurons, the UPR increases the protein expression of CHOP and caspase 12 resulting in marked apoptotic neuronal death [2,3,4]. ER-evoked CHOP-induced apoptosis has been reported to disrupt hippocampal synapses resulting in cognitive deficits. In the context of ER stress, PERK/GRP78/CHOP pathway plays a central role in mediating enhanced neuronal degeneration and apoptosis in CRS-exposed animals [3,4,11]. In fact, GRP78, a heat shock signal chaperone/transcription factor, is crucial in initiating the UPR and ER functioning [3]. In the same regard, CHOP, also known as the growth-arrest and DNA-damage inducible gene 153 (GADD153), has been identified as a transcriptional factor activated by ER stress resulting in the activation of the apoptotic pathway [39]. In line with these data, the present work demonstrated the activation of PERK/GRP78/CHOP axis that was evidenced by increased protein expression of iNOS, PERK, GRP78, and CHOP signals. Herein, magnesium sulfate inhibited PERK/GRP78/CHOP signaling in the hippocampus and suppressed ER stress, prompting better behavioral outcomes. The functional link between exaggerated ER stress and brain neurotransmission has been previously highlighted. In perspective, chronic ER stress has been reported to switch on spontaneous excitatory glutaminergic neurotransmission, an event that can be alleviated by NMDA receptor blockade [40]. This is in accord with the well-reported NMDA-receptor-blocking features of magnesium sulfate that were established in rodent models of eclampsia-like seizure [16] and focal cerebral ischemia [18]. Moreover, the present findings of ER stress mitigation and associated inhibition of PERK/GRP78/CHOP signaling by magnesium sulfate confirm the competence of NMDA receptor blockade to attenuate ER stress. In a rodent model of excitotoxic brain damage and brain ischemia, magnesium sulfate also demonstrated marked neuroprotection by dampening neuronal death and excitotoxic neurotransmission [16,18]. Moreover, dampening neuronal apoptosis by magnesium sulfate has been reported to mediate its protection in a mouse model of cerebral palsy [36]. 

## 4. Materials and Methods

### 4.1. Chemicals

Magnesium sulfate was purchased from Sigma-Aldrich Chemical Co (St. Louis, MO, USA). The highest analytical grade was selected for the remaining chemicals used in the current work.

### 4.2. Animals

Adult male albino rats (200–250 g; the Egyptian Drug Authority (EDA) animal house, Giza, Egypt) were used for the current set of experiments. Animal adaptation was performed for 2 weeks before the commencement of any experimental procedures. All rats were provided standard laboratory chow and water ad libitum under standard laboratory conditions (21–24 °C temperature, 12 h diurnal/nocturnal cycle, and 40–60% relative humidity). The Institutional Research Ethics Committee approved the current experimental protocol (NODCAR/I/45/2022). The care and handling of animals were applied in accordance with the ARRIVE guidelines and U.K. Animals Act, 1986. 

### 4.3. Animal Experiments

**Figure 11:**
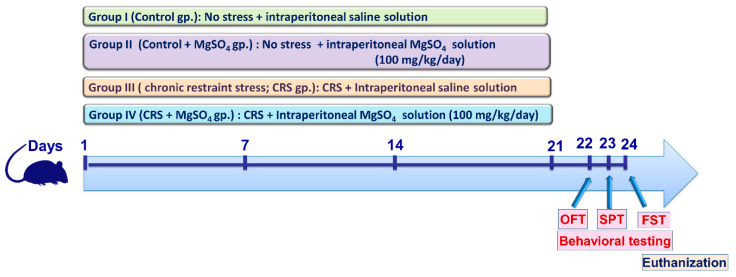
Schematic illustration of the experimental study design. At the end of the experimental period, the behavioral testing was applied starting from the least stressful test in the following order: (1) open-field test (OFT), (2) sucrose preference test (SPT), and (3) forced swimming test (FST), respectively. CRS, chronic restraint stress; MgSO_4_, magnesium sulfate.

In the current experimental protocol, 40 adult male Wistar rats were randomized into four experimental groups (*n* = 10 rats/each group). An outline of the experimental protocol is described in Figure 11. Group 1 (Control group): Rats that received a daily intraperitoneal dose of saline for 21 consecutive days without exposure to stress. Group 2 (Control + MgSO_4_ group): Rats that received magnesium sulfate (MgSO_4;_ 100 mg/kg/day; i.p.) for 21 consecutive days without exposure to stress. Group 3 (chronic restraint stress (CRS) group): Rats that were restrained for 6 h/day and received a daily intraperitoneal dose of saline for 21 consecutive days. Group 4 (CRS + MgSO4 group): Rats that were restrained for 6 h/day and received a daily intraperitoneal dose of MgSO_4_ (100 mg/kg/day) for 21 consecutive days.

The current CRS experimental protocol is in accordance with previous studies [2,41,42]. The selected dose of magnesium sulfate is consistent with the previous literature [43] and is in harmony with the clinically utilized dose for the management of pre-eclampsia in humans [44]. 

At the end of the experimental period, the behavioral tests were executed on days 22–24 (Figure 11). On the 24th day, anesthesia of the animals was carried out using sodium phenobarbital (150 mg/kg, i.p.) [45], and blood was withdrawn by an intracardiac puncture for serum separation. Then, euthanization was applied by decapitation and the 2 hippocampi were carefully dissected and stored at −80 °C for the biochemical measurements. To this end, the hippocampi were homogenized in ice-cold phosphate-buffered saline (pH 7.4) or RIPA buffer/protease inhibitor cocktail (ELISA assays) [46]. Moreover, the brains of 4 randomly selected animals from each group were placed in formol-saline for the histopathological examination. 

### 4.4. CRS Procedures

Restraint stress was applied for 6 h using restraining tubes every day till the end of the 21 days at random times during the diurnal phase of the diurnal-nocturnal cycle. The restraining tubes measured 9 cm wide × 24 cm long × 6 cm high. Restriction of the animal’s head and limb movement was applied by adjusting the length of the restraining tubes. To avoid exposure of the control and CRS + MgSO_4_ groups to stress, the animals of these groups were kept in another separate room with no contact with stressed animals. 

### 4.5. Behavioral Assessment

After the third week, the behavioral testing was executed starting from the least stressful to the most stressful test [23]. Hence, the following order was conducted: (1) open-field test on day 22, (2) sucrose preference test on day 23, and (3) forced swimming test on day 24, respectively (Figure 11). 

#### 4.5.1. Open-Field Test

The motor activity of the animals was assessed using the open-field test (OFT) [2,47]. The OFT apparatus is a 60 × 60 × 20 cm wooden box which is divided into 25 equal squares. The test was executed in a quiet room under dim white light to track animal behavior. Each animal was observed carefully for 3 min after placing it in the central square. The wooden box was cleaned with 70% ethyl alcohol after each individual trial. The study recorded the time taken by the rat to start a movement (latency time to leave the central square), ambulation, grooming, and rearing during the 3 min period.

#### 4.5.2. Sucrose Preference Test

The sucrose preference test (SPT) is utilized to examine anhedonia, which is an essential symptom in depression [48]. Prior to doing the SPT, the rats received 2 identical bottles of sucrose solution (1%) as the only source for drinking for 72 h as an acclimatization period. Before the testing day, all rats were deprived of drinking solution for 16 h. On the testing day, each animal was housed individually in the cage and 2 drinking bottles were placed, namely, water (100 g—weight) and 1% sucrose solution (100 g—weight), and the animal was free to choose to drink from the 2 bottles. To avoid potential side preference in drinking behavior, the position of the 2 bottles was altered every 6 h. After a constant time, the 2 drinking bottles were re-weighed and the weight difference was recorded. The following formula was used to calculate the sucrose preference %: (sucrose solution consumption (g) × 100)/(sucrose solution consumption (g) + water consumption (g)) [49].

#### 4.5.3. Forced Swimming Test

Using the method of Posolt and co-workers [50], the anti-depressant potential of magnesium sulfate was examined using the forced swimming test (FST) which examines the despair component of depression [51]. To this end, we used a cylinder tank (40 cm height × 22 cm diameter) filled with water at ambient room temperature (25°C) to the 25 cm mark. The test was applied for 5 min and the time of swimming to reach the tank wall was recorded. Moreover, the span where the animal did not swim was regarded as the immobility time. The water was changed after each trial in order to side-step confusing results.

#### 4.5.4. Z-Score Normalization of Behavioral Data

The Z-scores are mathematical tools with the advantage of allowing mean-normalization of the obtained data within studies, thereby providing a robust measure of the impact of magnesium sulfate on depression-relevant endpoints across the three tests used (open-field test, sucrose preference test, and forced swimming test) [20]. The Z-scores are standardized by the control group mean and control group standard deviation. The Z-scores were calculated by the following equation Z = X − µ/σ where X is the individual raw data, µ is the mean of the control group, and σ is the standard deviation of the control group. 

### 4.6. Measurement of Hippocampal Neurotransmitters

The content of serotonin (5-HT; MyBioSource Inc., San Diego, CA, USA, Cat. # MBS2700308), dopamine (DA; MyBioSource Inc., San Diego, CA, USA, Cat. # MBS7214676), and norepinephrine (NE; LifeSpan Biosciences, Inc., Seattle, WA, USA, Cat. # LS-F28027) was measured using rat-specific ELISA kits as instructed by the manufacturer [52]. The levels of the neurotransmitters were expressed as ng/g tissue. The optical intensity of the final color was read at 450 nm.

### 4.7. Measurement of Serum Corticosterone

To assess corticosterone levels, serum separating tubes were used for collecting the blood samples which were allowed to clot for 2 h at 25 °C, and then centrifuged at 1000× *g* for 15 min. Then, serum was assayed using rat-specific corticosterone ELISA kit (Wuhan Fine Biotech. Co., Ltd., Wuhan, China, Cat. # ER0859). The optical intensity of the final color was read at 450 nm.

### 4.8. Measurement of Inflammatory Markers

Rat-specific ELISA kits were used for the determination of hippocampal interleukin 1 beta (IL-1β) and tumor necrosis factor-alpha (TNF-α) levels (MyBioSource Inc., San Diego, CA, USA; Cat. # MBS825017 and Cat. # MBS355371, respectively). The optical intensity of the final color was read at 450 nm.

### 4.9. Measurement of 8-OHdG

MyBioSource kit was utilized to measure the hippocampal levels of 8-hydroxy-2′-deoxyguanosine (8-OHdG) according to the manufacturer’s instructions (MyBioSource; San Diego, CA, USA, Cat. # MBS267513). The optical intensity of the final color was read at 450 nm. 

### 4.10. Western Blot Analysis of iNOS/ PERK/GRP78/CHOP Axis

An immunoblotting protocol was used for examining iNOS/PERK/GRP-78/CHOP axis as described [53,54]. Briefly, homogenization of the hippocampus tissue was applied in a cold lysis buffer (Tris-HCL [50 mmol/L; pH 8.0], SDS [0.1% w/v], NP40 [1% v/v], NaCl [150 mmol/L], sodium deoxycholate [0.5% w/v], and phenylmethylsulfonylfluoride [0.5 mmol/L]). The lysate was centrifuged at 10,000× *g* and the supernatant was collected. The protein lysates (50 μg/lane) were subjected to SDS-PAGE and the separated proteins were transferred into a nitrocellulose membrane (Bio-Rad, Hercules, CA, USA). Following membrane blocking with non-fat dried milk in TBST, primary antibody incubation was applied overnight at 4 °C using specific antibodies against iNOS (Abcam, Waltham, MA, USA, Cat. # ab 178945), eNOS (Cat. # 32027), CHOP (Cat. # 2895), PERK (Cat. # 3192), and GRP78 (Cat. # 3183) at 1:1,000 dilution (Cell Signalling Technology, Beverly, MA, USA). After washing, the membranes were incubated with HRP-tagged secondary antibody (1:10,000, Cell Signalling Technology, Beverly, MA, USA) for 1 h at room temperature. Finally, protein visualization was performed using an enhanced chemiluminescence kit (ECL plus; Amersham, Arlington Heights, IL, USA) and band intensity quantification was applied using Molecular Analyst Software (Bio-Rad, Hercules, CA, USA).

### 4.11. Histopathological Examination and Neuropathological Damage Scoring

Rat brain autopsy samples were collected from each experimental group (*n* = 4), then fixed for 24 h in 10% formalin saline and embedded in paraffin wax [55]. The blocks of paraffin wax were sliced into 4 µm thick coronal sections using a sledge microtome. On glass slides, the obtained tissue sections were collected, then deparaffinized, stained with hematoxylin and eosin (H & E), and inspected under a light electric light microscope (Leica Microsystems, Wetzlar, Germany).

The neuropathological damage scores featuring the nuclear pyknosis were used to quantify the damage in the cortices and hippocampi of the animals based on a 0–4 score [22,23]. In this scoring system, a score of 0 indicated no specific lesion, a score of 1 indicated less than 10% affected area, a score of 2 indicated 20–30% affected area, a score of 3 indicated 40–60% affected area, and a score of 4 indicated more than 60% affected area. 

### 4.12. Statistical Analysis

The parametric values were expressed as mean ± standard deviation (S.D.). The homogeneity of data values was examined by the Shapiro–Wilk test. When the data were normally distributed around the mean (parametric data), statistical analysis was applied using one-way ANOVA, followed by Tukey’s test for multiple comparisons (GraphPad Prism Inc., San Diego, CA, USA). The values of the neuropathological damage scores (non-parametric data) were expressed as median with interquartile range and processed using the Kruskal–Wallis test followed by Dunn’s multiple comparison post-test. The minimum level of statistical significance was set at a *p*-value of <0.05. 

## 5. Conclusions

In conclusion, the promising role of magnesium sulfate in dampening CRS-evoked depression-like behavior was characterized by multi-pronged mechanisms. Notably, inhibition of the pro-inflammatory and pro-oxidant signals alongside the ER stress response and its linked PERK/GRP78/CHOP axis were essential in the favorable neuroprotection competence of magnesium sulfate. Further studies are encouraged to examine the detailed molecular mechanisms of neuroprotection exerted by magnesium sulfate in CRS-evoked depression-like behavior.

## Data Availability

Data are contained within the article.
